# The Causal Role of Bile Acids in Cancers of the Digestive System

**DOI:** 10.3390/biomedicines14030598

**Published:** 2026-03-08

**Authors:** Carol Bernstein, Harris Bernstein

**Affiliations:** Department of Cellular and Molecular Medicine, College of Medicine, University of Arizona, Tucson, AZ 85724, USA

**Keywords:** bile acids, cancer, gastrointestinal tract, reactive oxygen species

## Abstract

Bile acids are widely distributed in the human gastrointestinal tract. A literature review indicates that bile acids may have a role in initiating cancers in every organ of the digestive system. The estimated number of new digestive system cancers world-wide in 2022 was about 5 million. In the particular case of colon cancer, secondary bile acids produced in response to a high fat diet disrupt colonic epithelial cell mitochondrial membranes. This disruption leads to the release of oxidative free radicals that damage DNA, potentially leading to carcinogenic mutations. High levels of colonic bile acids may also alter the gut microbiome, with some bacteria causing inflammation and increased reactive oxygen species leading to DNA damage. Also, bile acids taken up by receptors on the surface of gastrointestinal tract cells can activate NF-kB. In turn, NF-kB may activate a super-enhancer at an oncogene. Bile acid reflux also plays a significant role in esophageal adenocarcinoma, stomach cancer and small intestine carcinogenesis. In addition, cancers of the pancreas, liver, and biliary tract can be caused by the constriction of the common bile duct leading to reflux of bile acids back into these organs. Gastroesophageal reflux involving bile acids may also contribute to hypopharyngeal squamous cell carcinogenesis. Thus, bile acids are a likely major contributory cause of cancer throughout the digestive tract.

## 1. Introduction

Bile acids act as emulsifiers during the digestion and absorption of dietary lipids and are also major signaling molecules [1]. However, high levels of bile acids may disrupt the mitochondrial outer cell membrane [2], causing generation of excessive reactive oxygen species, resulting in the induction of DNA damage, mutations, and apoptosis [3]. As indicated below, bile acids may also cause dysbiosis as well as the activation of NF-kB. O’Keefe et al. [4] showed that high concentrations of bile acids are detected in the feces of volunteers consuming a high fat diet.

## 2. Background

### 2.1. Where Do Cancers Come from?

As indicated in Table 1, an early report by Doll and Peto in 1981 [5] indicated the potential origins of all types of cancers in the United States (not just those in the digestive tract). A 2015 report by Blot and Tarone [6] additionally listed the recently recognized factors of obesity and infection (hepatitis viruses, *Helicobacter pylori*, etc.). It is evident from Table 1 that about 80% to 90% of cancers in the United States are caused by avoidable risk factors (ARFs).

From Table 1, diet, which controls bile acid release into the gastrointestinal tract, is an important factor in carcinogenesis.

### 2.2. How Does Cancer Arise?

Cancer arises from genetic mutations and epigenetic alterations that lead to dysregulated transcription in a cell [7]. (Epigenetic changes are alterations associated with the DNA that change the expression of the DNA).

Genetic mutations are one well-established major source of dysregulated transcription. Mechanisms by which bile acids generate mutations are described below in Section 2.3 and Section 2.4. Another more recently recognized major source of dysregulated transcription is from newly initiated super-enhancers. (Super-enhancers are clusters of DNA regulatory elements that strongly control the expression of key genes related to a cell’s identity). Bile acids, through the activation of NF-kB, appear to be a factor in the initiation of super-enhancers at oncogenes, as described below in Section 2.5.

### 2.3. Elevated Bile Acids Cause Oxidative DNA Damages, Leading to Mutations

The plasma membranes of cells are hardly affected by any of the bile acids, while the mitochondrial outer membrane structure is specifically modified by the cytotoxic secondary bile acid deoxycholic acid [2]. This leads to the release of reactive oxygen species [8]. At least 15 studies showed that bile acids caused DNA damage, likely through the actions of reactive oxygen species [9].

**Figure 1:**
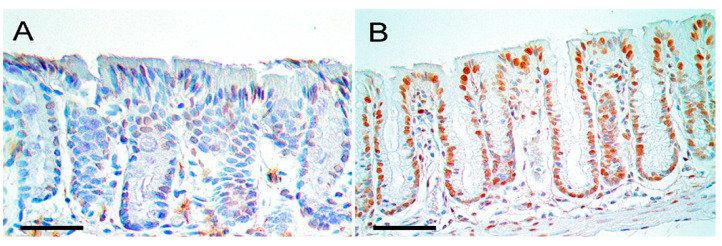
Colonic crypts in epithelium from two mice. (**A**) Tissue from a mouse fed a standard diet. (**B**) Tissue from a mouse fed a diet supplemented with deoxycholate. Scale bars: 50 μM. Cell nuclei are stained dark blue with hematoxylin (for nucleic acid) and immunostained red/brown for 8-OHdG. The level of 8-OHdG was graded in the nuclei of colonic crypt cells on a scale of 0–4. Mice fed a standard diet had crypt 8-OHdG at levels 0–2, and (**A**) is at level 1. Mice fed a diet supplemented with deoxycholate had 8-OHdG in colonic crypts at levels 3–4, and (**B**) is at level 4. Deoxycholate added to the mouse diet gave a concentration of deoxycholate in the mouse colon similar to the concentration in the colons of humans on a high fat diet. Modified from Chaya5260 CC-BY-SA-[4.0].

Figure 1B shows oxidative DNA damage (large presence of oxidized guanine: 8-OHdG) within the cellular nuclei of the colonic epithelium of a mouse fed for 6 months on a diet supplemented with deoxycholate (deoxycholate is the salt of deoxycholic acid). On a normal diet (see Figure 1A) there is little to no oxidized guanine in DNA in the nuclei of cells in the colonic epithelium. The mouse fed a diet supplemented with deoxycholate had colonic deoxycholate at a level similar to that of a human when the human is on a relatively high fat diet [10].

In general, substantial portions of the carcinogenicity of bile acids appear to be related to their detergent action on mitochondrial membranes [2]. This detergent action leads to disruption of the electron transport system employed in oxidative phosphorylation in the mitochondria and the consequent release of highly reactive oxidative free radical intermediates [8]. These oxidative free radicals are extremely damaging to DNA. Cells that are excessively exposed to bile acids will experience elevated DNA damage, and this results in DNA replication errors as well as DNA repair response errors, leading to mutations. Mutations occur randomly in a cell, reflecting the damaged DNA sequences that were mis-replicated or mis-repaired. These mutations can occur in locations that, for instance, inactivate tumor suppressor genes, or cause amplifications of oncogenes. As pointed out above, cancer arises from genetic and epigenetic alterations that lead to dysregulated transcription in a cell [7].

### 2.4. Elevated Bile Acids Cause Dysbiosis, Which Also Leads to DNA Damage and Mutation

Bile acids not only directly cause increased DNA damage and reactive oxygen species (ROS), but they may also cause dysbiosis. (Dysbiosis is an imbalance in the composition and function of the body’s microbial communities, typically referring to the gut microbiota).

In rats, high fat feeding increases primary and secondary bile acids. The increase in bile acids induces gut dysbiosis, resulting in mucosal inflammation [11].

When rats were orally supplemented with cholic acid, a taxonomic change in their gut microbiome was observed with an increase in bile acid-metabolizing phyla and a decrease in bile acid-intolerant bacteria. Specifically, this caused an increase in Firmicutes from 54% to 95% of the total microbiome and a decrease from 33% to less than 1% of Bacteroidetes and Actinobacteria [12].

As Mostafavi et al. [13] point out, while gut dysbiosis increases inflammation, it also expands the presence of ROS-producing bacteria, which further exacerbates inflammation. Gut dysbiosis reduces intestinal wall integrity and allows bacterial antigens and toxic materials to penetrate gut cells and enter the bloodstream, further amplifying ROS production.

The DNA damages caused by ROS are usually repaired by base excision repair. This repair is usually accurate, but can also infrequently give rise to mutations. ROS are mutagenic [14].

### 2.5. Newly Initiated Super-Enhancers Can Drive Dysregulated Transcription

**Figure 2:**
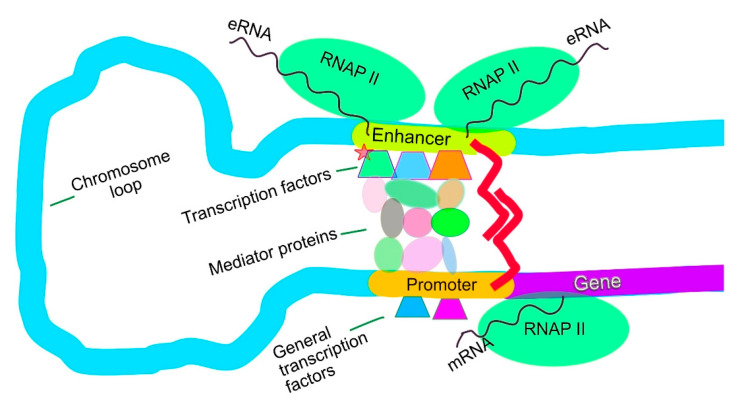
Typical enhancer. Transcription factors on the enhancer send signals through mediator proteins to the promoter to control transcription level of their target gene. The small star on a transcription factor indicates an activating phosphorylation. Bernstein0275—Own work CC BY-SA [4.0].

Super-enhancers are large genomic elements, comprising multiple constituent “typical enhancers” (see Figure 2) that work together to drive increased levels of gene transcription of the genes that they target.

**Figure 3:**
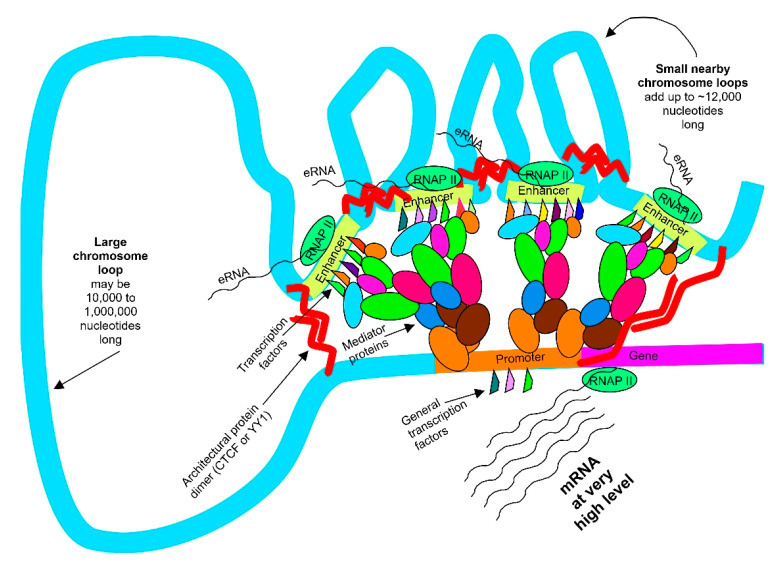
Super-enhancer with four constituent enhancers in a 12,000 nucleotide region of the genome. All four constituent enhancers are acting together to control the transcription level of their target gene.

There are generally a few hundred genes in a normal cell that are transcribed under the control of super-enhancers [15,16]. The super-enhancers (see Figure 3) often transcribe genes at a level about 4-fold higher (with some genes transcribed at about a 30-fold higher level) than the level controlled by typical enhancers for the same genes [16]. Super-enhancers often define the character of a cell’s identity.

Of the approximately 20,000 genes in the human genome, normal cells have 4000 to 9000 genes that are transcribed under the control of typical enhancers [15,16] (an activated enhancer is usually needed to start transcription of a gene). A typical enhancer is a stretch of DNA of about 700 to 1000 nucleotides in length with about 10 to 15 transcription factors bound to it. The enhancer loops around to a target gene. Then, the transcription factors bound to the enhancer send signals through a mediator complex (about 26 proteins in the complex) to the promoter of the target gene to start transcription (see Figure 3).

Super-enhancers usually consist of two to six typical constituent enhancers within a chromosomal region of 12,000 to 20,000 nucleotides in length. All the typical constituent enhancers simultaneously act in the super-enhancer to target a single gene (see Figure 3). In examinations of 18 cancer cells, there were 3 to 11 newly established super-enhancers which were driving increased expression of known oncogenes in those cancer cells. These newly up-activated oncogenes defined the cancer characteristics of those cancer cells [17].

**Figure 4:**
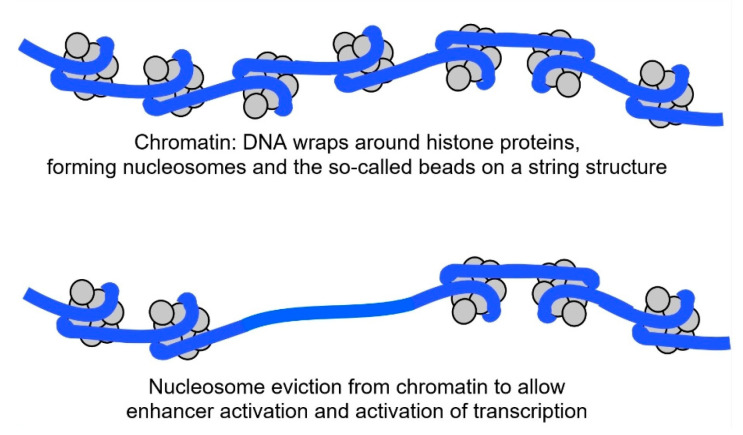
Top image: DNA in chromatin where DNA is wrapped around 8 histone proteins to form nucleosomes. Bottom image: Nucleosome has been evicted to allow transcription factors to attach to each of their DNA motifs.

To establish a new enhancer, a pioneer transcription factor is first needed to loosen the DNA from the histones it is wound around in a nucleosome. In the cell nucleus, NF-kB can act as a pioneer factor, loosening the DNA bound around the histone core [18] so that enzymes can access and evict the nucleosome (see Figure 4). Then, other transcription factors can bind to the bare area and create an active enhancer.

In the nucleus, Nf-kB can act as a pioneer factor, loosening the DNA bound around the histone core so that other transcription factors can also bind and create an activated enhancer [18]. The bile acid deoxycholate activates NF-kB, sending it to the nucleus [19]. This may have a signaling role in initiating super-enhancers that promote carcinogenesis [20].

## 3. Bile Acids in the Gastrointestinal System

**Figure 5:**
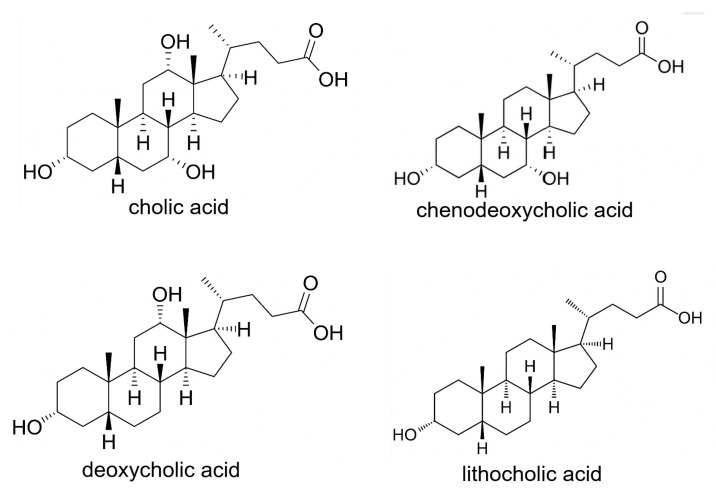
The primary bile acids, cholic acid and chenodeoxycholic acid, and the secondary bile acids, deoxycholic acid and lithocholic acid.

Bile acids are made by hepatocytes, which constitute 80% of the cells in the liver [21]. Hepatocytes act on cholesterol to create the primary bile acids, cholic acid and chenodeoxycholic acid [22]. Cholic acid and chenodeoxycholic acid are steroid molecules (steroids have three six member rings and one five member ring [23] (see Figure 5). The primary bile acids are conjugated with taurine or glycine which increase their solubility in an aqueous medium [24]. After conjugation, the primary bile acids have a hydrophilic side and a hydrophobic side, so they can adsorb to lipids on one side and use their other side to stay soluble in aqueous medium during digestion [24]. This promotes the digestion of lipids.

**Figure 6:**
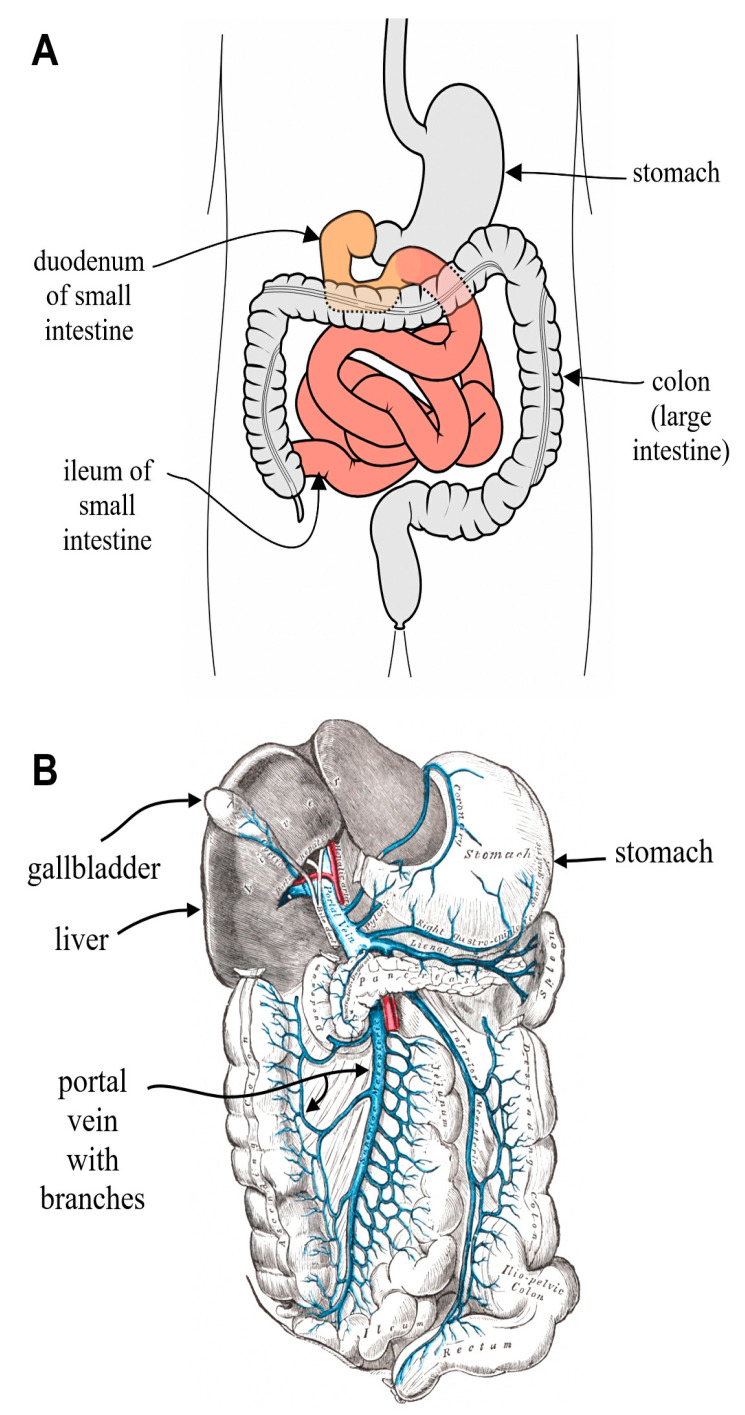
(**A**) Part of GI tract showing duodenum and ileum of small intestine. (**B**) Showing portal vein with its many branches accessing small intestine and colon.

In the hepatocyte, bile acids are at a concentration of 1–2 µM [24]. Hepatocytes conjugate bile acids, as they are produced, with taurine or glycine. The bile acids are rapidly transported into the biliary canaliculus to a concentration of 1000 µM (1 mM) and from there into the gallbladder where they become concentrated to 3–6 mM [25]. In the ggallbladder bile acids are both conjugated and integrated into micelles. Micelles are circular structures with bile acids complexed with cholesterol and phosphatidylcholine [26]. Bile acids within their micelles are exported from the gagallbladderhrough the common bile duct into the duodenum of the small intestine (for location of duodenum see Figure 6A) in response to a fatty meal. The bile acids exported to the duodenum are conjugated with taurine or glycine, as well as being largely within micelles. This greatly reduces their carcinogenicity [27].

The bile acids in the micelles adsorb to the lipids in the food, allowing the lipids to be taken up by the small intestine, and then about 95% of the bile acids are absorbed in the ileum, the lower small intestine (see Figure 6A). From the ileum, the reabsorbed bile acids are sent to the portal vein (see Figure 6B) and passed back into the liver.

When fasting, bile acids in the portal vein are at about 14 µM [28]. About 5% of the bile acids in the ileum are not absorbed and are passed through to the colon. The resulting level of bile acids in colonic contents is 0.6 mM (600 μΜ) [29]. Bile acids in the colon undergo bacterial transformation, primarily through removal of the conjugated taurine or glycine (deconjugation) and dehydroxylation, leading to the formation of the secondary bile acids (see Figure 5). Cholic acid is transformed into deoxycholic acid and chenodeoxycholic acid becomes lithocholic acid. When deconjugated in the colon, the secondary bile acids, deoxycholic acid and lithocholic acid, become cytotoxic and carcinogenic [27]. Overall, bile acids are at relatively low levels in the colon (see Table 2), but the deconjugated secondary bile acids in the colon are very carcinogenic [27].

Deoxycholic acid is reabsorbed in the colon and sent to the portal vein and from there into the liver (see Figure 6B), but lithocholic acid is largely removed through the feces. Because the bile acid pool is recirculated an average of six to eight times a day, and each time cholic acid is converted into deoxycholic acid, the final bile acid pool consists of about 40–50% cholic acid, 30–40% chenodeoxycholic acid and 20% deoxycholic acid [30,31].

The conjugated bile salts in the gallbladder are in micelles along with lecithin, and the micelles carry cholesterol with them. The amount of bile salts (in micelles) that are released into the small intestinal duodenum depends on the level of fat in a meal [32]. For the locations of the ggallbladder duodenum, etc., see Figure 2.

The “bile acid pool” is composed of all of the bile acids in circulation in the enterohepatic circulation. This includes the bile acids formed in the liver (<1%), in the intestine (small intestine and colon) (∼85–90%), and in the gallbladder (∼10–15%) [30].

Bile acids are also spilled over from the portal vein to the systemic circulation [30]. Fasting serum bile acid levels in the systemic circulation are about 2.4 µM [28,33]. After a meal, within 15–60 min, serum bile acids increase in concentration to 5.2 µM. Similarly, after a meal, the concentration of bile acids in the portal vein (through which bile acids taken up from the ileum and colon are returned to the liver) increases from 14 µM to a median of 43 µM [28].

## 4. Frequencies of Incidence and Mortality Due to Gastrointestinal System Cancers

In the US, the estimated number of new digestive system cancer cases in 2024 for both sexes was 353,820 [34], and estimated deaths was 174,320 [34]. Digestive system cancers may occur in any part of the digestive tract including the esophagus, the stomach, the small intestine, the colon and rectum, the liver, the gallbladder, the biliary tract and the pancreas [35]. Cancers of the digestive system accounted for about 18% of all US cancer cases arising in 2024 and about 28% of all cancer deaths [34]. World-wide, the estimated number of new cases and deaths for cancers of the colorectum, stomach, liver, esophagus and pancreas in 2022 was 4,957,675 new cases and 3,223,794 deaths [36].

**Figure 7:**
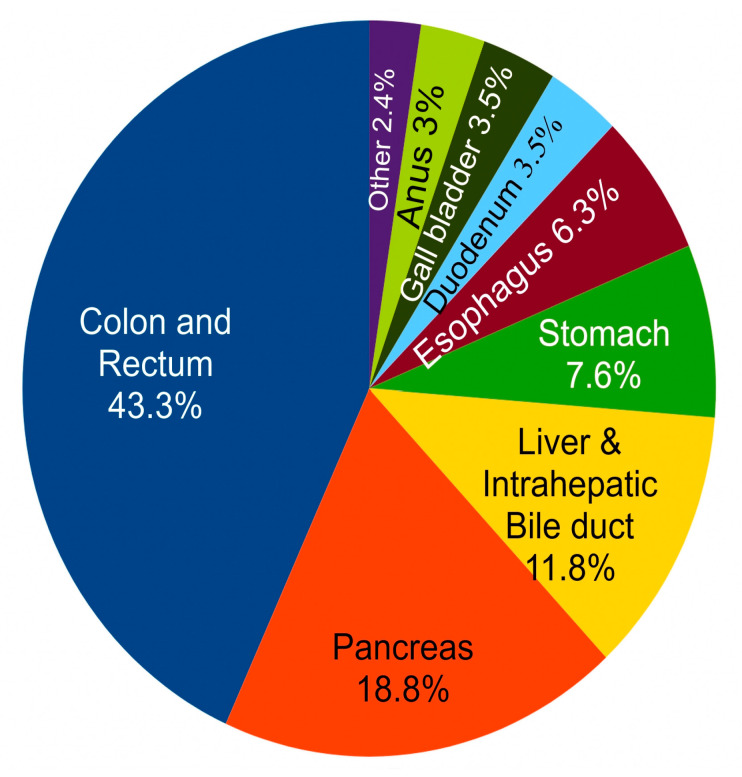
Percent incidence (USA) of different GI cancers.

Figure 7 shows the relative incidence of different cancers in the gastrointestinal (GI) tract. It is clear that the largest portion (43%) of cancers in the GI tract occurs in the colon and rectum.

## 5. Roles of Elevated Bile Acids in Gastrointestinal System Cancers

In the following sections the evidence for the role of bile acids in cancers of each of the organs of the digestive system is described.

### 5.1. Esophageal Cancer

Barrett’s esophagus is a precancerous condition in which the lining of the esophagus (see Figure 2) is damaged by acid reflux. This causes the esophageal cells to change from normal squamous epithelium (flat, scale-like cells) to columnar epithelium (tall, column-shaped cells) embedded with goblet cells (cup-shaped cells). Esophageal adencarcinoma arising from Barrett’s esophagus metaplasia is linked to reflux esophagitis (increased bile acids in the esophagus due to reflux) [37]. In 2024 in the US there were an estimated 22,370 new cases of cancer of the esophagus and 15,130 deaths [34]. World-wide in 2022, the estimated numbers of new cases and deaths for cancers of the esophagus were 510,716 new cases and 445,129 deaths [36].

About 8.1% of Barrett’s esophagus patients progress to cancer [38]. Barrett’s esophagus has been attributed mainly to gastroesophageal reflux disease leading to chronic inflammation of the esophagus [39].

Bile acids are present in gastroesophageal reflux into the esophagus and are implicated in neoplastic development in the esophagus [40]. Bile reflux contains unconjugated bile acids that include chenodeoxycholic acid and deoxycholic acid, likely contributing to the development of esophageal adenocarcinoma by inducing oxidative stress and DNA damage [41]. The secondary bile acid deoxycholic acid at doses of 100 μM and higher induces DNA damage in esophageal cells by a mechanism involving reactive oxygen species [42]. Secondary bile acids are potent DNA damaging agents because they disrupt mitochondrial membranes leading to the release of DNA damaging reactive oxygen species into the cytosol [40].

In a transgenic mouse model, the progression of Barrett’s esophagus to esophageal adenocarcinoma was accelerated by exposure to bile acids [39].

### 5.2. Stomach Cancer

In 2024 in the US the incidence of stomach cancer was 26,890 and there were 19,880 deaths [34]. World-wide in 2022 the estimated number of new cases and deaths for cancers of the stomach was 968,350 new cases and 659,853 deaths [36].

In addition to *Helicobacter pylori* infection, bile reflux is a likely factor in causing gastric intestinal metaplasia, which is a precancerous lesion leading to gastric cancer [43,44]. It was noted by Shi et al. [45] that smoking and drinking loosen the sphincter of pylorus, and that generates a retrograde biliary flow of bile from the duodenum to the stomach via a slack pylorus. Smoking and drinking increase the risk of gastric cancer [46]. It was also noted that bile acids in gastric aspirates were present at a concentration of 1000 μM in patients with bile reflux gastritis (a condition associated with carcinogenesis). In healthy individuals, on the other hand, bile acids were only present in gastric aspirates at a level of 30 μM [47]. Tatsugami et al. [48] followed 357 patients infected with *Helicobacter pylori* for up to three years. There was a strong positive correlation between levels of gastric bile acids and histologic gastritis, intestinal metaplasia (a precursor to gastric cancer) and progression to gastric cancer. This indicated a role of bile acids in gastric carcinogenesis in addition to infection by *H. pylori*.

He et al. [49] noted that *H. pylori* infection has a major role in causing gastric intestinal metaplasia, the precursor to gastric cancer development. However, they summarized the likely equally important contributions by refluxed bile acids to intestinal metaplasia. This included the negative effects of bile acids on gastric cell membranes, dysbiosis in the stomach and epigenetic alterations in the miRNA expression profile. In addition, they noted that bile acids cause the activation of NF-kB as well as the interaction with the bile acid receptors FXR and TGR5 to cause strongly altered expressions of markers of intestinal metaplasia (increased CDX2 and decreased SOX2).

Dysregulation of bile acids and gut bacteria along with the resulting impairment of the gastric mucosa appear to be central factors in the pathogenesis of precancerous lesions [44]. Bile reflux alters the composition of the gastric bacteria [50]. Conjugated bile acids are increased in the stomach of people with bile reflux gastritis and gastric cancer [51]. Irritation of the gastric mucosa over a long period by bile reflux likely has a role in gastric carcinogenesis [52]. Bile acids, a component of bile reflux, appear to be a causal factor in gastric carcinogenesis [52]. Altered bile acid metabolism (particularly upregulation of the bile acid deoxycholic acid) when linked to iron deficiency promotes *Helicobacter pylori*-induced inflammation-driven gastric carcinogenesis [53,54]. In a National Retrospective Cohort Analysis, it was found that the use of bile acid sequestrant modification is protective against cardia (upper part of stomach) and non-cardia (main body of stomach) gastric cancer [55].

A carcinogenic interaction was demonstrated between bile acids and *Helicobacter pylori* in a mouse model of gastric cancer [53,54].

### 5.3. Small Intestine Cancer

The length of the small intestine is about 75 percent of the total length of the gastrointestinal tract [56]. About 50% of the adenomas occurring in the small intestine arise in the duodenum even though this location comprises only 4 percent of the length of the small intestine. Such adenomas arise mainly close to the ampulla of Vater. The ampulla of Vater is the outlet of the common bile duct where bile acids are released into the small intestine [57]. In 2024 in the US there were an estimated 12,440 new cases of cancer of the small intestine and 2090 deaths [18].

A prospective study involving human subjects observed a markedly elevated risk for carcinoid tumors of the small intestine associated with dietary intake of saturated fat [58]. Such a diet is associated with increased bile acid exposure.

Cholecystectomy, a surgical procedure to remove the gallbladder, alters the flow of bile to the small intestine and increases the risk of small intestinal adenocarcimoma [59]. This risk declines with increasing distance from the common bile duct.

### 5.4. Colon and Rectum Cancer

The International Agency for Research on Cancer (IARC) has classified processed meat as a group I carcinogen. This classification is based on the IARC finding of sufficient evidence that consumption of processed meat by humans causes colorectal cancer [60,61,62]). Processed meat is generally high in fat. In 2024 in the US there were an estimated 152,810 new cases of cancer of the colon and rectum and 53,010 deaths [34]. World-wide in 2022, the estimated numbers of new cases and deaths for cancers of the colorectum were 1,926,116 new cases and 903,859 deaths [36].

A high fat diet is associated with high levels of bile acids that appear to increase the risk of colorectal cancer [4,35,63]. In particular, the bile acid deoxycholic acid is increased in the colonic contents of humans on a high fat diet [35,63]. Also, fecal concentrations of bile acids, particularly deoxycholic acid, are higher in populations that have a high incidence of colorectal cancer [35,63]. Bacteria such as *Fusobacterium nucleatum*, *Streptococcus bovis*, *Helicobacter pylori*, *Bacteriodes fragilis* and *Clostridium septicum* produce secondary bile acids from primary bile salts and these may cause inflammation and DNA damage leading to colorectal carcinogenesis [64].

A meta-analysis on the relationship of fecal bile acid concentrations to the development and progression of colorectal cancer showed that high fecal concentrations of the bile acids cholic acid and chenodeoxycholic acid are associated with an increased risk and increased incidence of colorectal cancer [65].

In one prospective study, baseline serum was collected 30 years prior to diagnoses of colorectal cancer [66]. It was found that, among women, there was a strong association between serum concentrations of bile acids (collected and analyzed 30 years previously) and increased colorectal cancer risk [66]. In a separate study, fecal concentrations of deoxycholic acid were dramatically increased after cholic acid administration, suggesting that the interaction between bile acids and the gut microbiota affects colon cancer progression [67]. In another prospective study it was found that prediagnostic plasma levels of certain conjugated primary and secondary bile acids (e.g., glycocholic acid, taurocholic acid, glycochenodeoxycholic acid, taurochenodeoxycholic acid, glycodeoxycholic acid, taurodeoxycholic acid) are positively associated with colon cancer risk [68].

#### 5.4.1. A Bile Acid-Based Mouse Model of Colon Cancer

A diet-related mouse model of colon cancer was devised that involved feeding wild-type mice a standard diet supplemented with deoxycholate. This produced a level of deoxycholate in the mouse colon comparable to that in the colon of humans eating a high fat diet [10,69]. Eight to ten months after initiating the diet, 45 to 56 percent of the mice developed colonic adenocarcinomas.

On the basis of histopathologic examination and the expression of specific markers, the colonic tumors in the mice were essentially identical to those in humans [10]. In humans, defective fields that surround colon cancers and the colon cancers themselves exhibit characteristic aberrant changes in molecular markers. The colonic tissues of the mice fed a diet containing deoxycholic acid showed similar changes in biomarkers. For instance, 8-OHdG in DNA was increased (see Figure 1, above), the DNA repair protein ERCC1 was decreased, the autophagy-related protein beclin-1 was increased, and, in the stem cell region at the base of crypts, there was a substantial nuclear localization of beta-catenin as well as an increase in cytoplasmic beta-catenin [10]. Aberrant activation of the Wnt/β-catenin signaling pathway leads to the accumulation of β-catenin in the nucleus, where it acts as an oncogenic transcription factor [70].

#### 5.4.2. Epigenetic Alterations in Colon Cancer Are More Frequent than Mutations

As described by Vogelstein et al. [71], an average colon cancer has one or two mutations in oncogenes and one to five mutations in tumor suppressor genes (together called “driver mutations”), and about 60 “passenger” mutations.

Epigenetic alterations, as distinct from mutations, change the protein expression of genes without changing the DNA sequence [72]. Epigenetic alterations are much more frequent in colon cancer than genetic (mutational) alterations.

**Figure 8:**
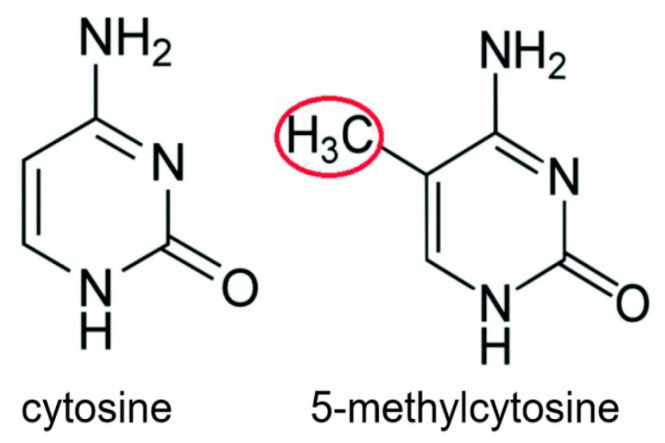
Cytosine and 5-methylcytosine. The red circle indicates the added methyl group.

In two studies, about 600 increased methylations of cytosines (see Figure 8) and about 300 decreased methylations of cytosines occurred in promoters of genes in colon cancers [73,74]. Decreased methylation of cytosine within the promoter of a gene is an epigenetic alteration that increases expression of the gene [75]. Methylation of cytosines in DNA can be reduced by increased presence of reactive oxygen species [76]. Bile acids at higher levels can cause increased release of reactive oxygen species from the mitochondria [8], thereby likely increasing epigenetic upregulated expression of some genes.

A second type of epigenetic alteration is the increased/decreased production of particular microRNAs (miRNAs). Each miRNA typically regulates the expression of several hundred genes [77]. The miRNAs either cleave their target messenger RNAs (mRNAs) or they attach to their target mRNAs and prevent them from coding for proteins [78]. A total of 164 miRNAs were found to be significantly altered in various colorectal cancers. About 2/3 of miRNAs were increased while about 1/3 were reduced [79]. Methylation alteration of cytosines within the promoters of genes with embedded miRNAs is one of the mechanisms for changing miRNA expression [80]. Oxidative damage to a guanine (caused by high levels of bile acids) can cause the demethylation of an adjacent cytosine [76], and this may be within promoters that control miRNAs, possibly causing the upregulation of the affected miRNA.

### 5.5. Liver Cancer (Hepatocellular Carcinoma)

Hepatocellular carcinoma is an aggressive and prevalent liver malignancy. In 2024 in the US there were an estimated 41,630 new cases of cancer of the liver and intrahepatic bile duct and 29,840 deaths [34]. World-wide in 2022, the estimated numbers of new cases and deaths for cancers of the liver were 865,269 new cases and 757,984 deaths [36].

Several recent studies have presented evidence that bile acids have an etiologic role in hepatocellular carcinoma [81,82,83]. Cholestasis is a condition where the flow of bile from the liver is reduced or blocked. A central factor in the pathogenesis of cholestasis-induced liver injury is hepatic accumulation of bile acids, and excessive cytotoxic bile acids in the liver may lead to liver fibrosis and cirrhosis, and then progression to liver cancer [81]. In a prospective study, it was found that the risk of developing hepatocellular carcinoma is associated with higher levels of the major circulating bile acids that were measured several years prior to tumor diagnosis [84]. Another large prospective cohort study found that serum levels of total bile acids are associated with an increased risk of hepatocellular carcinoma in patients with cirrhosis [85]. Also, evidence was reviewed indicating that alterations in bile acids by the human gut microbiome contribute to hepatocarcinogenesis [86]. The microbiota in the gut that contribute to hepatocellular carcinoma seem to be distinct from those that cause colorectal cancer [87]. The homeostasis of bile acids is especially disturbed in individuals with a history of alcohol intake [88]. Alcohol appears to trigger bile acid disequilibrium to initiate and promote hepatocellular carcinoma progression [88].

Using a mouse model, it was found that dysregulated hepatic bile acids collaboratively promote liver carcinogenesis [89].

### 5.6. Gallbladder Removal (Cholecystectomy) and Cancer

In vertebrates, the gallbladder is a small organ where bile is stored and concentrated prior to being released into the small intestine after a high fat meal. Bile is produced in the liver and transferred to the gallbladder via the common hepatic duct. The gallbladder can then release bile via the common bile duct into the duodenum (see Figure 6), where it assists in the digestion of fats.

The major role of the gallbladder in humans is to protect cells of the gastrointestinal tract from the cytotoxic effects of some of the bile acids [90].

Thus, in a normal healthy individual, bile acids are primarily released from the gallbladder into the gastrointestinal tract in response to a fatty meal. The bile acids are passed down the gastrointestinal tract in a short period during digestion. The primary bile acids (cholic acid and chenodeoxycholic acid) and the secondary bile acids (deoxycholic acid and lithocholic acid) do not cause an increase in cell death if the exposure to each of the four bile acids is 2 h and the level of exposure is less than 200 μM [91]. Bile acids may be at less than 200 μM when mixed with food during digestion.

However, if the gallbladder is removed (“cholecystectomy,” often due to gallstones), then the gastrointestinal tract is more continuously bathed in bile acids. Under continuous exposure, cholic acid is not cytotoxic, even at high levels of 500 μM. However, under continuous exposure, chenodeoxycholic acid, deoxycholic acid and lithocholic acid are cytotoxic at almost any level, with increasing cytoxicity at increasing levels of exposure [91]. Lithocholic acid, however, is not a major factor in toxicity since it is excreted in the feces. However, if multiple fatty meals are eaten, then more bile acids are circulated through the colon, where non-toxic cholic acid is converted by bacterial action to cytotoxic deoxycholic acid [92]. Also, higher levels of circulation of the bile acid pool occur after cholecystectomy [93]. Then, as seen in Table 3 (adapted from [31]), with cholecystectomy, cholic acid is reduced and replaced with the cytotoxic deoxycholic acid.

Cytotoxic deoxycholic acid concentration is doubled in the bile acid pool in patients with a cholecystectomy, compared to deoxycholic acid concentration in healthy subjects (Table 3) [31].

Cholecystectomy is associated with increases in at least four different gastrointestinal cancers. (1) Cholecystectomy was significantly associated with increased right-sided colon cancer, especially in the cecum, the ascending colon and/or the hepatic flexure, but not in the transverse, descending, or sigmoid colon [94]. (2) Similarly, from five pooled studies, there is a 15% increased risk of gastric cancer after cholecystectomy [95]. (3) Again, in an examination of the records of 278,460 cholecystectomized patients in Sweden, cholecystectomy increased the risk of small intestinal cancer. The relative risk was 1.77 compared to individuals without cholecystectomy. The risk decreased with distance from the common bile duct [59]. (4) Among 42,098 patients in Denmark who had a cholecystectomy, there was a relative risk of pancreatic cancer of 1.42 [96].

As pointed out by Chaing and Ferrell [30], there is spillover of bile acids into the serum. With a cholecystectomy there would be relatively higher levels of the cytotoxic deoxycholic acid in the serum. The deoxycholic acid would be carried in the blood circulation to many organs of the body. Choi et al. [97] examined the data from 123,295 individuals who had a cholecystectomy and a matched set of 123,295 individuals who did not have a cholecystectomy in the database of the Korean National Health Insurance Service. They evaluated the records for occurrence of any of 23 cancers in a followup period of an average of 4.6 years. Those who had a cholecystectomy had an increased risk of a subsequent cancer in leukemia, colon, liver, pancreas, biliary tract, thyroid, pharynx and oral cavity.

### 5.7. Gallbladder and Biliary Tract Cancer

In 2024 in the US there were an estimated 12,350 new cases of cancer of the gallbladder and other biliary structures and 4530 deaths [34].

Cholangiocarcinoma (bile duct cancer) is a type of cancer that comprises mutated epithelial cells that originate in the bile duct. The activities of gut microbiota and bile acids are linked and have crucial roles in the pathogenesis and progression of cholangiocarcinoma [98]. Primary sclerosing cholangitis is a chronic liver disease where bile ducts, both inside and outside the liver, become inflamed and scarred, leading to narrowing or blockage. Primary biliary cholangitis is a chronic autoimmune disease where the body’s immune system mistakenly attacks and destroys the bile ducts in the liver. Progression to bile duct cancer is promoted both by primary sclerosing cholangitis and primary biliary cholangitis, both of which are associated with cholestasis where the flow of bile from the liver is reduced or blocked [98]. The tumors in about 25% of patients with biliary tract cancer have some form of deficiency in DNA damage repair [99].

Normal function of the gallbladder ordinarily protects against carcinogenesis. This protective function is indicated by observations that removal of the gallbladder (cholecystectomy) causes an increased subsequent risk of cancer. A systematic review and meta-analysis of eighteen studies concluded that cholecystectomy had a harmful effect on the risk of right-sided colon cancer [94]. An additional recent study reported that subsequent to a cholecystectomy there is a significantly increased total cancer risk [97].

Bile salts from the cytoplasm of hepatocytes are transferred into the bile canaliculi by the bile salt export pump. When bile salt export is deficient due to a mutation in the gene *ABCB11* (*ATP-binding cassette, subfamily B member 11*) that encodes the bile export pump, the result can be intrahepatic toxic accumulation of bile salts. Individuals who have such mutations experience an increased incidence of hepatocellular carcinoma or cholaniocarcinoma (bile duct cancer) [100].

### 5.8. Pancreatic Cancer

In 2024 in the US there were an estimated 66,440 new pancreas cancer cases and 51,750 deaths [34]. World-wide in 2022, the estimated numbers of new cases and deaths for cancers of the pancreas were 510,566 new cases and 467,005 deaths [36]. The most common form of pancreatic cancer is pancreatic ductal adenocarcinoma [101]. Bile acids are strongly implicated in the growth and progression of pancreatic cancer [101].

Patients with pancreatic cancer often also have biliary obstruction and elevated bile acids, which contribute to causing these tumors [102]. Sixty percent of the cases arise in the pancreatic head, 15% in the body or tail, and 20% involve the gland diffusely [103]. Bile acid reflux caused by biliary obstruction appears to affect the epithelial cells or acinars from which pancreatic adenocarcinoma is derived [104]. When the composition of bile acids in bile (extracted from the common bile duct) of patients with adenocarcinoma of the pancreas was compared with the composition of bile acids from patients with benign disease, it was found that the concentration of the unconjugated bile acid cholic acid in the malignant group was significantly higher than in the benign group [105]. Dysregulation of the interaction of bile acids with gut microbiota appears to play a role in pancreatic cancer progression [106]. Bile acids appear to promote the carcinogenic process in pancreatic ductal adenocarcinoma cells by a process in which increased expression of the MUC4 protein plays an important role [107]. MUC4 protein is a major constituent of mucus, the viscous secretion that covers epithelial surfaces. The majority of the risk factors for developing pancreatic cancer include smoking, a high fat diet and alcohol intake, each leading to high bile acid secretion [104]

In hamsters, bile-reflux into the pancreatic ducts is observed to be associated with intraductal papillary carcinoma development [108].

### 5.9. Oral Cavity and Pharynx

Although often treated separately from the “digestive system”, the oral cavity and pharynx are also subject to bile acid-induced cancer. In the US, the estimated numbers of new cancer cases in 2024 at sites in the oral cavity and pharynx for both sexes were 58,450 and estimated deaths was 12,230 [34]. Oral cavity and pharynx cancers may occur in the tongue, mouth and pharynx. In 2024 these cancers accounted for about 2.9% of all US cancer cases and about 2.1% of all cancer deaths [109].

Bile acids appear to be a significant causal factor in human hypopharyngeal squamous cell carcinoma [110]. In gastroesophageal refluxate, bile acids are often present and as a consequence can cause inflammatory and neoplastic alterations in the upper aerodigestive tract [111]. The STAT3 protein appears to have a significant role in bile reflux-induced molecular events linked to hypoparyngeal carcinogenesis [112]. STAT3 protein is a transcription factor that can bind to DNA and influence gene transcription and gene expression in such cellular processes as cell growth, differentiation and survival.

In a mouse study, it was found that acidic bile refluxate produces oxidative DNA damage, which can lead to progressive mutagenic effects and hypopharyngeal squamous cell carcinogenesis [110].

## 6. Clinical Relevance

As pointed out by Ridlon et al. [27], deoxycholate is a metabolite whose serum and colonic levels correlate with carcinogenesis. Ridlon et al. summarize the properties of deoxycholate with respect to membrane perturbing and the activation of cellular signaling pathways associated with cell proliferation and apoptosis resistance. These pathways provide mechanisms for the long-term environmental risk of neoplasia.

A human study was conducted with 40 healthy individuals, age range 18–35 years, over a six month period [113]. This study showed that individuals on a controlled high fat diet (40% of calories from soybean oil) had increased deoxycholate in their fecal samples. The individuals on the high fat diet also had considerably altered colonic microbiota, compared to similar groups of individuals on a low fat (20% fat) or moderate fat (30%) diet. The groups on low or moderate fat diets had no increases in their colonic deoxycholate.

Americans in 1990 had diets averaging 34.1% of calories from fat, down from an average of 36.4% of calories from fat in 1972 [114]. Still, 34.1% of calories from fat on average would imply that many Americans are eating diets with more than 40% of calories from fat. A diet of more than 40% of calories from fat would likely raise their level of cytotoxic colonic deoxycholate. The type of fat would also be important. Cancer risk would be reduced by 8% if 10% of saturated fats were replaced by polyunsaturated fats [115].

Overall, patients may be able to reduce their risk of cancer if they reduce the calories consumed per day from fat. The American Cancer Society [116] lists ways to reduce the consumption of fat in the diet. They recommend reading labels on commercial foods and choosing foods where 30% or less of calories comes from fat. As an example, whole milk has about 50% of its calories from fat. The American Cancer Society recommends using 1% milk which has about 20% of calories from fat. Cancer risk may be reduced further if foods with high levels of saturated fats, such as beef fat (which has about 50% saturated fat in its fat composistion) are avoided. Beef could be replaced with chicken or fish. Chicken and fish fats have about 30% saturated fats in their fat composition.

## 7. Summary

Although bile acids do not directly damage DNA, we indicated in the Introduction that bile acids are a likely major indirect source of DNA damage in the cell by disrupting mitochondrial membranes leading to the release of oxidative free radicals that are highly DNA damaging [2]. A comparative and comprehensive study was conducted on the impact of cytoprotective and cytotoxic bile acids on the membrane structure of individual cellular compartments [2]. It was found that the outer membranes of mitochondria are the likely main target of the cytotoxic bile acid deoxycholic acid. The cell’s mitochondrial membranes were found to be more sensitive to deoxycholic acid-induced structural changes than the cell’s plasma membrane. The disruption of mitochondrial outer membranes induced by bile acids causes the release of reactive oxygen species that may damage the cell’s DNA.

Bile acids, at increased levels, may also cause dysbiosis. This dysbiosis causes inflammation, releasing reactive oxygen species, and some newly prominent bacteria also produce reactive oxygen species, further damaging DNA and causing mutations.

Bile acids interact with receptors in epithelial cell membranes, causing the activation of NF-kB and sending NF-kB to the nucleus. NF-kB can act as a pioneer transcription factor, and may initiate the formation of a super-enhancer at an oncogene, leading to its increased expression and stimulating cancer development.

Bile acids serve an important nutritional function, namely in the utilization of dietary fat as a significant food source. However, the evidence reviewed above indicates that when exposure of the digestive tract to bile acids is excessive over extended periods it can cause DNA damage and altered gene expression, leading to cancer.

For each of the organs of the digestive tract, we have summarized above the evidence that elevated exposure to bile acids contributes to cancer development. In the particular case of colorectal cancer, substantial evidence based on numerous studies supports a causal relation of bile acid activity to carcinogenesis. For the other organs of the digestive tract, the evidence is also generally supportive of a significant role of bile acids in carcinogenesis. Thus, we consider it reasonable to propose that increased levels of bile acids are a major cause of cancer throughout the digestive system.

## Figures and Tables

**Table 1:** Percentages of cancer mortality caused by avoidable risk factors (ARFs) in the United States (numbers are approximatr due to rounding).

Major Factors	Percentage of Deaths Caused by ARFs (85% of Total Deaths) [5]	Percentage of Deaths Caused by ARFs (80–90% of Total Deaths) [6]
Diet	35	35
Tobacco	30	30
Obesity	-	6.5
Reproductive and Sexual	7	7
Occupational	4	4
Alcohol	3	3
Infection	-	10
Geophysical (sunlight, radon, etc.)	2	4
Pollution	2	1–5
Medicines	<1	1–3
Industrial products (non-occupational)	<1	-
Food additives	<1	1

**Table 2:** Levels of bile acids and states of conjugation at locations in the gastrointestinal tract.

Location	Level (mMolar)
Hepatocytes (all conjugated)	0.001–0.002
Biliary canaliculus (all conjugated)	1
Gallbladder (all conjugated and in micelles)	6
Portal vein—fasting (mixed conjugated and unconjugated)	14
Colon (all unconjugated)	0.6

**Table 3:** Pool sizes of different bile acids in healthy controls and patients with a cholecystectomy.

Pool Size (μM per Kilogram)		Bile Acid
Healthy Controls	Cholecystectomy	
48.7	28.2	Cholic
31.1	33.0	Chenodeoxycholic
21.4	42.5	Deoxycholic
1.2	11.3	Lithocholic

## Data Availability

No new data were created or analyzed in this study. Data sharing is not applicable to this article.
